# Automated assessments of circumferential strain from cine CMR correlate with LVEF declines in cancer patients early after receipt of cardio-toxic chemotherapy

**DOI:** 10.1186/s12968-017-0373-3

**Published:** 2017-08-02

**Authors:** Marie-Pierre Jolly, Jennifer H. Jordan, Giselle C. Meléndez, Gary R. McNeal, Ralph B. D’Agostino, W. Gregory Hundley

**Affiliations:** 1 Medical Imaging Technologies, Siemens Healthineers, 755 College Road East, Princeton, NJ 08540-6632 USA; 2 Department of Internal Medicine, Section on Cardiovascular Medicine, Wake Forest University School of Medicine, Medical Center Blvd, Winston-Salem, NC 27157 USA; 3 Department of Pathology, Section on Comparative Medicine, Wake Forest University School of Medicine, Winston-Salem, NC USA; 4 Cardiovascular MR R&D, Siemens Healthineers, Chicago, IL USA; 5 Biostatistical Sciences, Wake Forest School of Medicine, Winston-Salem, NC USA

**Keywords:** Cardiovascular magnetic resonance, Strain, Chemotherapy, Cancer therapy cardiotoxicity

## Abstract

**Background:**

In patients with cancer receiving potentially cardio-toxic chemotherapy, measurements of left ventricular (LV) circumferential or longitudinal strain are often used clinically to identify myocardial dysfunction. Using a new software algorithm, we sought to determine in individuals receiving treatment for cancer the association between automated assessments of LV mean mid-wall circumferential strain and conventional measures of LV ejection fraction (EF) both obtained from cardiovascular magnetic resonance (CMR) cine balanced steady-state free-precession (bSSFP) white-blood acquisitions.

**Methods:**

Before and 3 months after initiating treatment with potentially cardio-toxic chemotherapy, 72 individuals (aged 54 ± 14 years with breast cancer [39%], lymphoma [49%], or sarcoma [12%]) underwent serial CMR cine bSSFP assessments of LV volumes and EF, and mean mid-wall circumferential strain determined from these same cine images as well as from additional tagged CMR images. On the cine images, assessments of strain were obtained using the newly developed deformation-based segmentation algorithm. Assessments of LV volumes/EF from the cine images and strain from tagged CMR were accomplished using commercially available software. All measures were analyzed in a blinded fashion independent of one another.

**Results:**

Acceptable measures for the automated assessments of mean mid-wall circumferential strain from the cine images were obtained in 142 of 144 visits (98.6%) with an overall analysis time averaging 6:47 ± 1:06 min. The results from these automated measures averaged −18.8 ± 2.9 at baseline and −17.6 ± 3.1 at 3 months (*p* = 0.001). Left ventricular EF declined slightly from 65 ± 7% at baseline to 62 ± 7% at 3 months (*p* = 0.0002). The correlation between strain from cine imaging and LVEF was *r* = −0.61 (*p* < 0.0001). In addition, the 3-month changes in LV strain and LVEF were correlated (*r* = −0.49; *p* < 0.0001). The correlation between cine and tagged derived assessments of strain was *r* = 0.23; *p* = 0.01.

**Conclusions:**

Automated measures of LV mean mid-wall circumferential strain can be obtained in 6¾ minutes from cine bSSFP LV short-axis images (used concurrently to assess LV volumes and EF) in 98.6% of patients receiving treatment for cancer with potentially cardio-toxic chemotherapy. These cine derived measures of circumferential strain correlate with early subclinical declines in LVEF.

## Background

Chemotherapeutic regimens, including those incorporating anthracyclines or trastuzumab, for the treatment for cancer can induce myo-cellular injury that promotes declines in left ventricular (LV) function [1]. Undetected, these declines in LV function can lead to the development of heart failure and increased cardiovascular (CV) morbidity and mortality. As a result, noninvasive imaging techniques are often used to detect early deteriorations in LV ejection fraction (LVEF) or myocardial strain so that chemotherapeutic regimens may be modified or cardio-protective medications instituted in order to avert the development of irreversible LV dysfunction or untoward CV events [2, 3].

Cardiovascular magnetic resonance (CMR) has been utilized to identify early adverse declines in both LVEF and mean mid-wall circumferential strain in patients receiving potentially cardio-toxic chemotherapy [3]. In these studies, CMR myocardial strain was measured utilizing techniques that incorporate myocardial tissue tagging [3]. In many situations however, “tag fading” and poor image quality results in incomplete tracking of the myocardium [4]. As a consequence, more recently, investigators have measured myocardial strain directly from cine CMR images without the application of tissue tags [5–7]. Direct measures of strain from cine acquisitions avoid the additional time necessary to acquire or issues associated with analysis of tagged images.

The purpose of this study was to determine the feasibility and performance metrics of an automated method to measure mean LV mid-wall circumferential strain from cine images acquired in individuals before and 3 months after receipt of potentially cardio-toxic chemotherapy. A second objective of this study was to determine in these same study participants the association between cine assessments of myocardial strain and a) LVEF, b) tagged assessments of myocardial strain, and c) other risk factors for CV events.

## Methods

### Study population and design

This prospective study was approved by the Wake Forest Health Sciences Institutional Review Board and all participants provided written, witnessed informed consent. Eligible participants scheduled to receive potentially cardio-toxic chemotherapy were consecutively recruited over a 5 year period from the Comprehensive Cancer Center and the hematology/oncology outpatient clinics of Wake Forest Health Sciences. Participants were ineligible if they had a contraindication to CMR (e.g., implanted metal or electronic devices). CMR images were acquired for all patients before and 3 months after initiating cancer treatment to assess LV volumes, LVEF, and myocardial strain. The CMR imaging was conducted each morning prior to the scheduled administration of chemotherapy. In addition to CMR, we also collected blood samples to measure serum troponin levels. Finally, the participants completed a modified Minnesota Living with Heart Failure Questionnaire (MLHFQ) [8], before and 3 month after initiating cancer treatment.

### Cardiovascular magnetic resonance

CMR images were acquired using a 1.5 T Siemens MAGNETOM Avanto (Siemens Healthcare, Erlangen, Germany). Cine balanced steady state free precession (bSSFP) imaging was performed using breath-hold retrospective ECG gating to acquire a stack of short axis slices as well as 2 long axis views (2-chamber and 4-chamber). Short axis images were acquired with a slice thickness of 8 mm (with 2 mm gap), a 38 × 30 cm field-of-view (FOV), a 160 × 120 matrix, and a temporal resolution of 35–45 ms (with a reconstruction of 40 to 60 frames per RR interval). Long axis images were acquired with a slice thickness of 6 mm, a 36 × 30 cm FOV, a 192 × 160 matrix, and a temporal resolution of 20–30 ms (with a reconstruction of 50 to 60 frames per RR interval). Parallel imaging (GRAPPA) was utilized with an acceleration factor of 2 to 3.

Tagged imaging was performed according to previously published methods using the spatial modulation of magnetization technique at the middle papillary muscle level of the LV short axis as identified in the 4-chamber view [9]. Imaging parameters included a 34 cm FOV, a 10 ms TR, a 4 ms TE, a 20 degree flip angle, an 8 mm slice thickness, and a 256 × 160 matrix. After acquisition, all images were analyzed in a blinded fashion with analysts blinded to participant identifiers, cancer therapy dosing, and the image analysis results from other portions of the study (e.g., strain, LV volumes, or tagging).

### Automatic segmentation of the myocardium for cine strain assessments

**Fig. 1:**
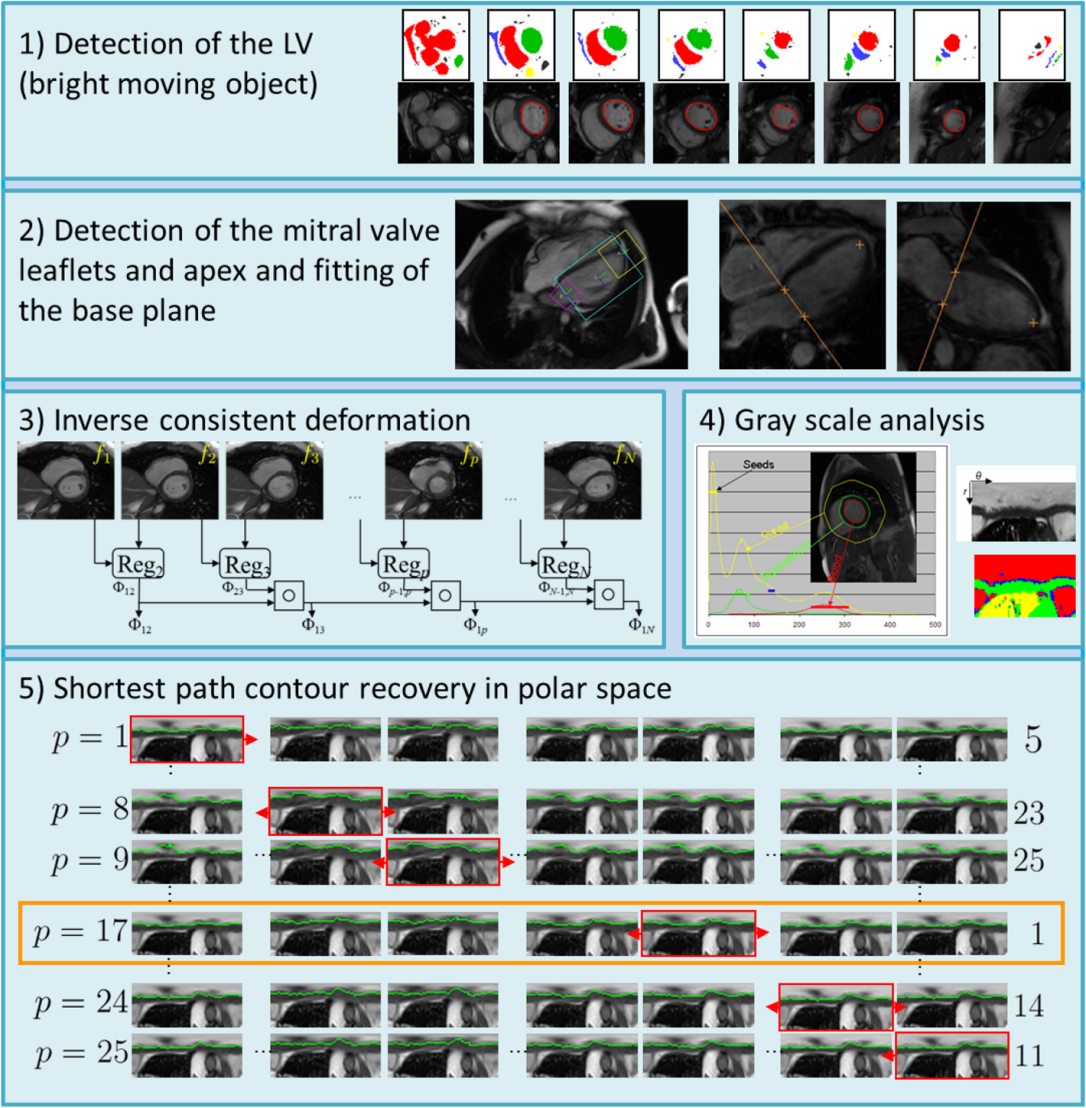
Automatic algorithm to segment the LV blood pool cavity and myocardium. Each box shows a step in the algorithm. *1*) Detection of the blood pool as the bright moving object. *2*) Detection of the mitral valve anchor points in the long axis images and fitting of the mitral valve base plane over time. *3*) Registration of neighboring frames to establish deformation fields between the first frame and any other frame in the time series. *4*) Histogram analysis to determine the major regions in the image, namely, blood, myocardium, and lungs. *5*) Shortest path algorithm to recover a contour, endocardium or epicardium in polar space

The CMR cine images were processed using a prototype for automatic segmentation of the LV blood pool cavity and myocardium (Trufi Strain, Siemens Healthcare, Medical Imaging Technologies, Princeton, NJ, USA). The algorithm implemented in the software is summarized in Fig. 1. The algorithm included the following steps.

**Fig. 2:**
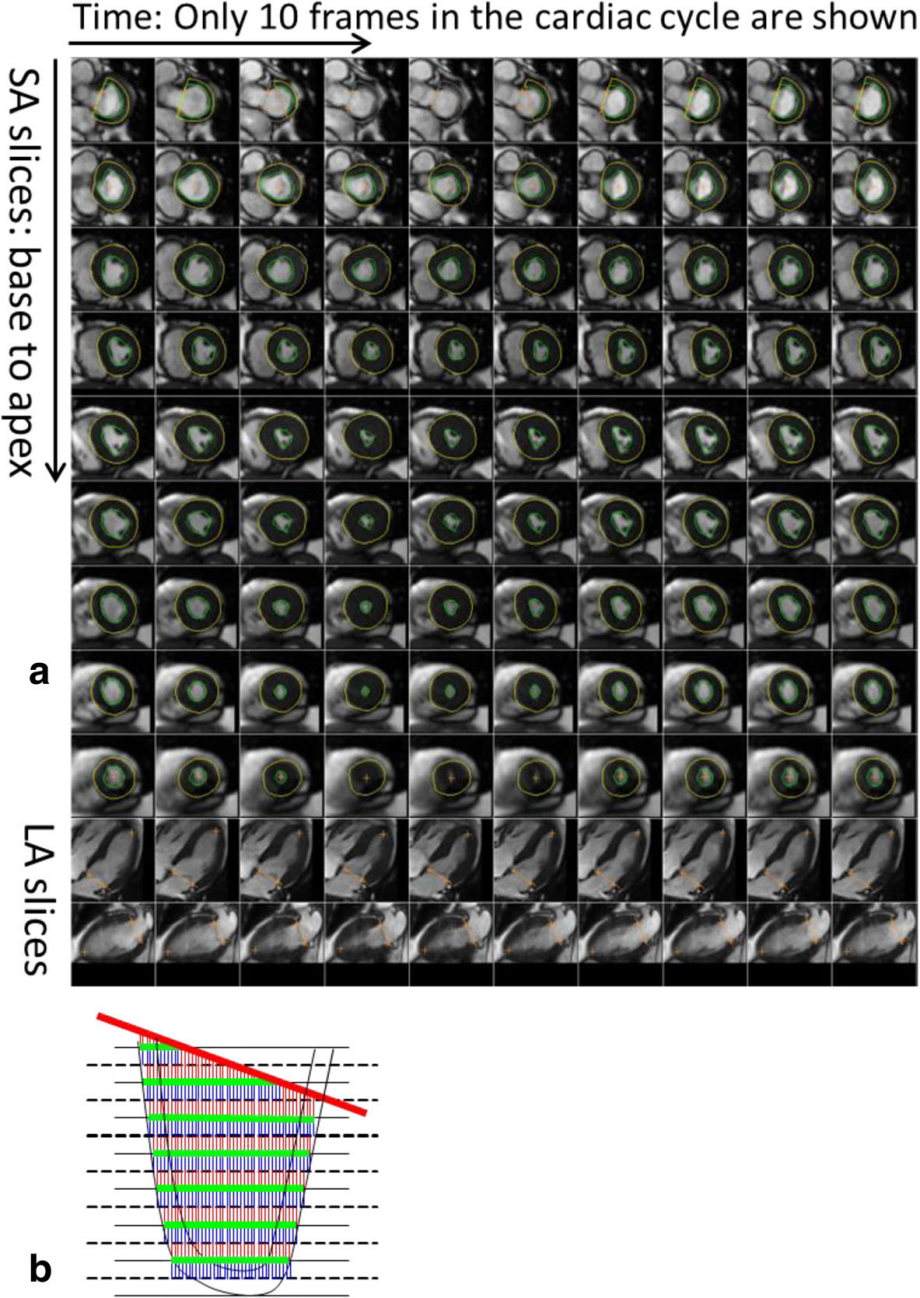
Description of the automatic process to calculate left ventricular (LV) volume and strain. As shown, **a**) the LV endocardium and epicardium are automatically contoured on the LV short axis slices. The mitral valve base plane is detected on the long axis slices of the left ventricle and projected onto the LV short axis slices. **b** To calculate the LV volumes, slices are stacked and contoured areas are summed up. At the same time, the mitral valve base plane is intersected with the stack of slices to remove portions of the volume that are above the base plane and inside the left atrium

1. The center of the LV cavity and the right ventricle insertion points were detected and used to hypothesize the position and radius for the left ventricle in each slice. As previously described [10], the blood pool was detected as a moving, bright, round object which overlaps with the detected centers in the various slices.
2. The mitral valve insertion points were detected on the long axis slices and a plane was fitted to the four mitral valve points detected on the 2-chamber and 4-chamber views.
3. A dense deformation field was defined between images in a slice to enforce temporal consistency in the recovered LV endocardial and epicardial contours throughout the cardiac cycle. The registration algorithm calculated a deformation field between two images at different cardiac phases by minimizing the local cross correlation and solving a partial differential equation using gradient descent. Since it was not feasible to calculate a deformation field between all possible pairs of images in a cardiac slice, the registration algorithm was extended to become inverse consistent. We used the efficient updating scheme proposed previously to update both the deformation field and its inverse at each step of the gradient descent [11]. In addition, two passes of the registration algorithm were performed, the first one to register all the images to the first phase and the second one to register all the images to the last phase. The final deformation fields were obtained by averaging the deformation fields from the two passes to ensure that the motion pattern was circular.
4. Histograms of the LV regions were analyzed to generate gray level properties for the different regions of interest (blood, myocardium, air, and partial volume). These properties were then used to calculate the possibility of each pixel representing an edge between two regions.
5. The endocardial and epicardial contours were recovered in each slice. The images were converted to polar space and each pixel was assigned a cost based on the image contrast between neighboring pixels (image gradient). A contour was then recovered in a polar image using Dijkstra’s shortest path algorithm. This contour was propagated to the other images in the slice using the deformation fields. The cost of all the contours was calculated based on the cost of the individual contours. This was repeated for each individual image in the slice. The combination of contours with the smallest cost resulted in the final segmentation [12, 13]. An example of automatic myocardium segmentation is shown in Fig. 2a.

Once the contours were obtained, the volume of the LV cavity was calculated by stacking up the areas of the contours in the different slices. In addition, we excluded segments above the mitral valve base plane corresponding to the left atrium as depicted in Fig. 2b.

**Fig. 3:**
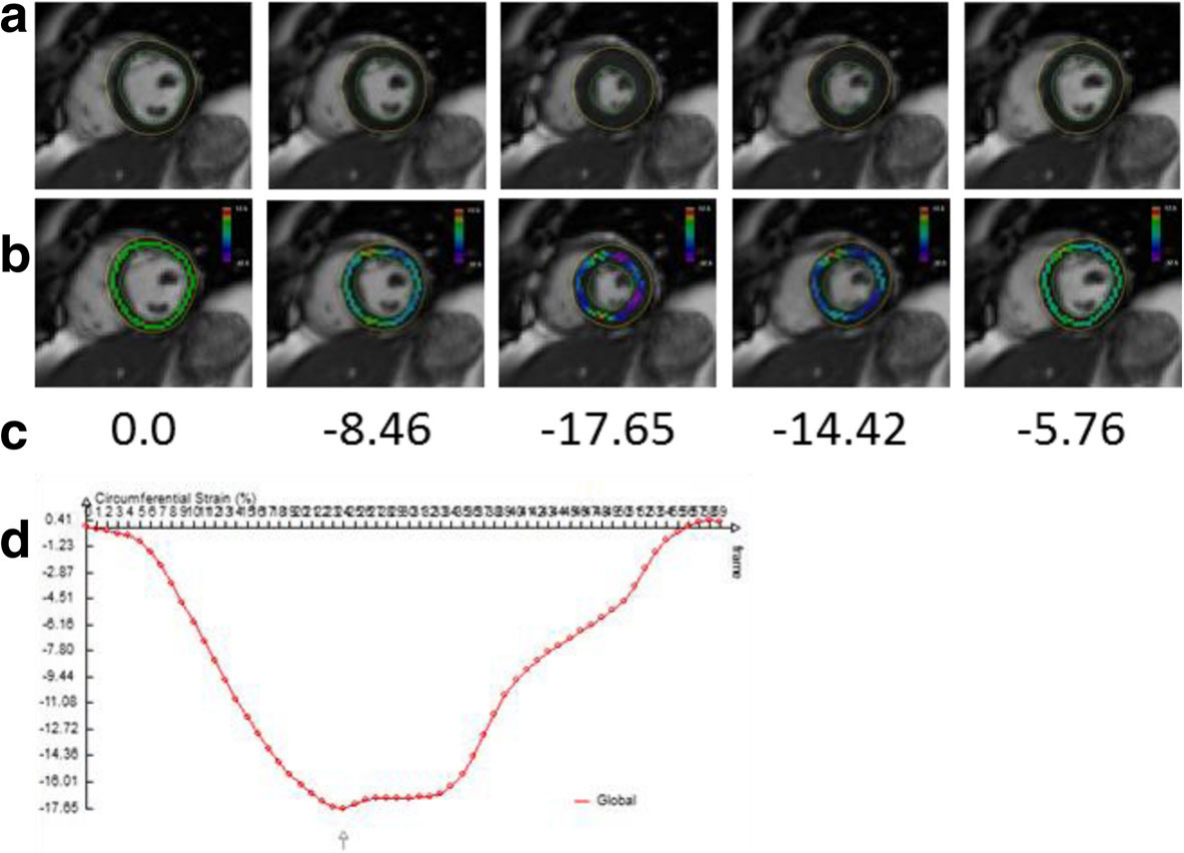
Mid-ventricular mid-myocardial circumferential strain calculations: These panels display **a**) images over time with contours; **b**) images over time with strain on mid-myocardial pixels; **c**) average values of the circumferential strain for the images; and **d**) the average mid-ventricular mid-myocardial circumferential strain over the 60 frames of the cardiac cycle

The deformation fields were then used to calculate Lagrangian mid-wall circumferential strain values within the LV myocardium. The Lagrangian strain tensor was expressed directly in terms of the gradients of the displacement vectors. The x and y strain were converted into a local polar coordinate system to calculate radial and circumferential strains at every pixel. The circumferential strain was averaged over the pixels that were on the centerline between the LV endocardial and epicardial surfaces. The LV mid-ventricular slice was identified as the slice in the middle of the automatically contoured slices. Figure 3 shows the pixels that are used to calculate the average circumferential strain for a few images over the cardiac cycle as well as the LV mid-ventricle mid-myocardial average circumferential strain over the entire cardiac cycle.

### Manual measurement of left ventricular ejection fraction

The same set of cine slices was manually analyzed using QMASS (Medis, Leiden, The Netherlands) for the purpose of determining LV volumes and LVEF. An operator blinded to the patient and visit information manually outlined the endocardium and epicardium from the end-diastolic (ED) and end-systolic (ES) phases for both the baseline and 3-month visits for all patients. The ED and ES volumes (EDV and ESV) as well as the LVEF were calculated according to modified Simpson’s Rule Technique [3] from the manual contours.

### Manual measurement of left ventricular strain from the tagged images

Harmonic phase (HARP) analysis (Diagnosoft, Morrisville, North Carolina, USA) was used to measure the mid mean circumferential strain from grid-tagged CMR images. A mesh was traced around the epicardial and endocardial contour of the mid left ventricle in a frame during systole using the anteroseptal intersection of the right ventricular wall as a landmark. Eulerian circumferential shortening strain was computed at the mid-wall of each segment with peak baseline circumferential systolic strain subsequently measured on the time strain curves. Strain values were averaged using 6 segments (anterior, anteroseptal, septal, inferior, posterior, and lateral); with visual inspection, some segments could be dropped from the average if tracking of the tags throughout systole and early diastole could not be accomplished.

### Statistical analysis

Descriptive statistics were calculated for variables with means, standard deviations, and ranges for continuous variables, and counts and percentages for categorical variables. Next, 3-month changes were estimated for the parameters of interest (LV volumes, LVEF and strain). Paired t-tests were calculated for each measure. Two-sample t-tests were then examined to determine whether there were differences at each time point (baseline and 3-months) or in the 3-month change among patients with different characteristics including: anthracycline use, gender, hypertension, diabetes, previous coronary artery disease (CAD), smoking status, or use of angiotensin converting enzyme inhibitors, beta-blockers, or HMG-CoA reductase inhibitors (statins) for each outcome measure of interest. For measures with more than 2 categories (cancer type (breast, lymphoma, or sarcoma) or number of CV risk factors (0–3)), one-way analysis of variance (ANOVA) models were fit to determine the relationship between these risk factors and the strain values (baseline, 3-month, and 3-month change). Pearson correlations were then estimated between the patient’s age, the number of CV risk factors, and the cumulative dose of their anthracycline (in doxorubicin equivalents) with their strain measures. Next, Pearson correlations were calculated between the LV strain from cine imaging, the strain from tagged imaging, and the ejection fraction, for all patients and all visits. Also, Pearson correlations were calculated to examine the relationship between the 3-month change in LV strain with the 3-month change in LVEF. In addition, correlations between the LV strain, LVEF measures (at each time point and the 3-month differences) and cumulative doses of chemotherapy for each type of regimen were examined. A Fisher’s exact test was performed to determine whether there was a relationship between baseline to 3-month changes in LVEF (yes/no) with changes in strain (increase/decrease). We also examined whether the relationship between the 3-month change in LVEF (outcome) and baseline LV-strain (or 3-month change in LV strain) was modified when patient level risk factors (age, gender, hypertension, diabetes, CAD and smoking status) were included as covariates in a general linear model. Finally, we categorized the patients into 2 groups based on their LVEF change/value at 3 months. Patients whose LVEF dropped more than 10%, patients whose LVEF dropped to below 53% at 3 months, and patients whose LVEF dropped by more than 10 to below 53% were compared to those patients whose LVEF remained within normal limits. We compared continuous measures (LV strain, age) for those with LVEF drop (yes/no) using 2-sample t-tests and categorical variables (gender, hypertension, diabetes, smoking, CAD) using Fishers exact tests. All analyses were performed using SAS (SAS Institute, Cary, North Carolina, USA).

## Results

A total of 72 patients were enrolled in this study and a pair of CMR cine scans was available for each patient. Legends Table 1 summarizes the characteristics of the 72 participants and indicates their chemotherapy treatment with some participants receiving more than one potentially cardio-toxic chemotherapeutic regimen.

**Table 1:** Participant demographics

		Subjects (*N* = 72)
Age (years)		53.8 ± 14.2
Men		24 (33%)
Hypertension		26 (36%)
Diabetes		11 (15%)
CAD		4 (6%)
Smoker		8 (11%)
Cancer Type	Breast	28 (39%)
	Lymphoma	35 (49%)
	Sarcoma	9 (12%)
Chemotherapy type	Anthracycline	49 (68%)
	Antimicrotubule agents	48 (67%)
	Alkylating agents	56 (78%)
	Tyrasine-Kinase inhibitors	28 (39%)
	Antimetabolites	4 (6%)
Receipt of cardio-protective medications		24 (33%)

In 2 out of the 144 cases (1.4%), the automatic segmentation algorithm was not able to produce reasonable contours. It took 6:47 ± 1:06 min to automatically contour the myocardium and calculate the strain on the 142 datasets with automated analyses. In the other 2 cases, a semi-automated solution was performed. In this situation, the analysis times were 15:18 min and 12:36 min, respectively for each case. As such, strain data from the white blood cine bSSFP images was available for all 72 participants at both visits. The tagging generated strain assessments could only be measured on 64 and 61 patients at each time point, respectively (125 of 144 total measurements).

The difference between two independent repeated measures of the automated assessments of the cine strain measurements was 0 ± 0 (*R* = 1.0). There was no randomness in the automated algorithm determined measures of strain across all the study participants, thus, there was complete (100%) reproducibility. In the 2 cases when the resulting contours were not acceptable, and therefore drawn manually, the average difference in the strain values between the different experiments was 0.66 ± 0.57. To evaluate the reproducibility for gathering tagged derived strain assessments, we studied 8 subjects (4 men and 4 women; aged 41 to 49 years) with an LVEF >60% and no visible LV segmental wall motion abnormalities on 2 separate occasions within the same day. For the group, peak average mid-wall circumferential strain was −13 + 4% and −13 + 4% on exams 1 and 2, respectively (one of the individuals had known coronary arteriosclerosis). The correlation between the 2 measurements was excellent, y = 0.97× + 0.5, R2 = 0.98. These data demonstrate good reproducibility of the tagged derived strain measure.

The cine derived automated analysis of measures of strain were −18.8 ± 2.89 and −17.6 ± 3.08, respectively at the baseline and 3-month visit. The tagging derived strain values were −17.0 ± 2.58 and −16.5 ± 2.42 prior to and 3 months after initiating chemotherapy, respectively. Overall, the two different methods of measuring strain (cine white blood versus tagging) correlated weakly with one another (*r* = 0.23; *p* = 0.01). While both techniques for assessing strain indicated a deterioration (less negative value) over the 3-month sample interval, the correlation between the baseline to 3-month changes in the cine automated derived measures of strain and the tagging measures was not significant (*r* = 0.18; *p* = 0.18). The variability of the two different methods’ strain measures across the population were similar (as shown by both different methods exhibiting somewhat similar standard deviations of the respective measures collected within the cohort).

Table 2 provides the LV end-diastolic volume, end-systolic volume, LVEF and strain values at the baseline and the 3-month visits. As shown in Table 3, the cine white blood bSSFP automated analysis derived measures of mean mid-wall circumferential strain correlated with LVEF; and there was no correlation between tagging derived measures of strain and LVEF. Overall for the study, the procentual reduction in LVEF was 4.2 ± 10%. The average absolute reduction in LVEF was 3.0 ± 6.6%.

**Table 2:** Changes in CMR metrics between the baseline and 3-month visit

	Baseline Pre-Chemotherapy	3 Months After Chemotherapy	Change (*p*-value)	Pearson correlation from difference in strain (*p*-value)
EDV (ml)	130.22 ± 37.7	124.0 ± 37.3	−6.26 (0.024)	−0.34 (0.004)
ESV (ml)	46.54 ± 20.03	48.18 ± 20.93	1.63 (0.25)	0.09 (0.44)
EF (%)	65.11 ± 6.57	62.11 ± 7.02	−3.00 (0.0002)	−0.49 (<0.0001)
Strain	−18.81 ± 2.89	−17.58 ± 3.08	1.23 (0.001)	

*Abbreviations*: *EDV* end-diastolic volume, *ESV* end-systolic volume, *EF* ejection fraction

**Table 3:** Pearson correlation coefficients, number of observations, and significance of association (*p*-values) for measures of left ventricular cine derived strain, tagged derived strain, and ejection fraction

R (*N*, *p*-value)	Cine derived strain	Tagged derived strain	Ejection fraction
Cine derived strain	1.0 (144)	0.23 (125, 0.01)	−0.61 (144, <0.0001)
Tagged derived strain	0.23 (125, 0.01)	1.0 (125)	−0.09 (125, 0.30)
LVEF	−0.61 (144, <0.0001)	−0.09 (125, 0.30)	1.0 (144)

**Fig. 4:**
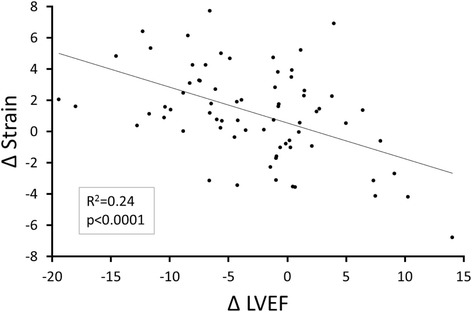
Correlation between 3-month change in strain and 3-month change in left ventricular (LV) ejection fraction. Change in left ventricular ejection fraction (x-axis) versus the change in LV strain on the y-axis; each point represents data from 1 participant. The regression line and correlation are shown

Three month changes in the cine white blood derived measures of LV myocardial strain correlated with 3-month changes in LVEF (*r* = −0.49, *p* < 0.0001; Fig. 4). As shown in Table 4, the majority of patients (54.2%) demonstrated a decrease in LVEF and a decrease in absolute value of the LV mean mid-wall circumferential strain measured automatically over the 3 months of chemotherapy treatment. Seventy-one percent (71%) of the participants exhibited a concordant change in LVEF and strain, either an increased LVEF and improved strain or a decreased LVEF and diminished strain.

**Table 4:** Frequency of increase or decrease in left ventricular ejection fraction and mean mid-wall circumferential strain derived from cine white blood images

Frequency (percent)	Strain absolute increase	Strain absolute decrease	
LVEF decrease	9 (12.5%)	39 (54.2%)	Fisher’s Exact Test
LVEF increase	12 (16.67%)	12 (16.67%)	*p* = 0.012

*Abbreviations*: *LVEF* left ventricular ejection fraction

When we examined the demographic variables (age and gender), co-morbidities (hypertension, diabetes, coronary artery disease, or smoking), type of cancer (breast, lymphoma, etc.), receipt of cardiovascular medications (e.g., beta blocker, statin), and use (yes/no) and dose of anthracyclines received, we found a negative correlation between age and baseline pre-cancer treatment cine white blood derived measures of mean mid-wall circumferential strain (*r* = −0.33, *p* = 0.005), but not with 3-month (*r* = −0.18, *p* = 0.13) or baseline to 3-month changes in strain (*r* = 0.13, *p* = 0.29).

We found a negative correlation between the dose of anthracycline and the change in LVEF (*r* = −0.30, *p* = 0.012). When comparing patients receiving anthracycline vs. patients receiving a different treatment, there was no difference in cine derived strain or strain change (*p* = 0.35–0.9), and no difference in LVEF before and after 3 months of chemotherapy. There was a difference in change in LVEF between the two populations. The patients receiving anthracycline had a drop in LVEF of −4.21 ± 6.13% while the others experienced little change in LVEF (−0.14 ± 6.65%, *p* = 0.013).

Table 5 shows the Pearson correlations between a change in cine derived strain or in LVEF and age, change in troponin levels, change in MLHFQ and the cumulative anthracycline dose. It can be seen that only the change in LVEF is weakly correlated to the anthracycline dose. There were no other significant associations between serum troponin, MLHFQ results, or treatment variables and 3-month changes in LV mean mid-wall circumferential strain (*p* > 0.15 for all comparisons).

**Table 5:** Pearson correlation coefficients, number of observations, and significance of association (*p*-values) for measures of LV cine derived strain and LVEF to age, changes in MLHFQ, change in troponin, and anthracycline dose

R *p*-value, *N*	Age	Change inMLHFQ	Change in troponin	Anthracycline Dose
Change in cine derived strain	0.127 0.29, 72	−0.058 0.63, 71	−0.017 0.89, 72	0.010 0.93, 72
Change in LVEF	−0.204 0.09, 72	−0.070 0.56, 71	0.012 0.92, 72	−0.295 0.01, 72

*Abbreviation*s: *MLHFQ* Minnesota living with heart failure questionnaire

Table 6 shows t-test associations between the change in cine derived strain or LVEF and gender and cardiovascular risk factors (gender, hypertension, diabetes, CAD, and smoking). Gender was associated with strain at baseline (women versus men, −19.5 and −17.5, respectively, *p* = 0.005) and at the 3-month follow-up visit (women versus men, −18.2 and −16.3, respectively, *p* = 0.009). However, as shown in Table 6, the 3-month change in mean mid-wall circumferential strain did not differ by gender. There were no significant correlation between CV risk factors and 3-month changes in LV mean mid-wall circumferential strain (*p* > 0.64 for all comparisons). After multivariable analyses, we found that after adjusting for age, gender, and cardiac risk factors, changes in cine derived strain associate with changes in LVEF (*p* < 0.0001).

**Table 6:** t-test associations between change in cine derived strain and change in ejection fraction compared to gender and risk factors

Mean in each group *t*-value *p*-value	Gender	Hypertension	Diabetes	Coronary artery disease	Smoking
Change in cine derived strain	M: 1.21 F: 1.24 *t* = 0.03 *p* = 0.98	Yes: 1.10 No: 1.45 *t* = −0.47 *p* = 0.64	Yes: 1.11 No: 1.25 *t* = 0.13 *p* = 0.89	Yes: 1.34 No: 1.22 *t* = −0.07 *p* = 0.94	Yes: 1.63 No: 1.24 *t* = −0.33 *p* = 0.74
Change in ejection fraction	M: −3.97 F: −2.51 *t* = 0.89 *p* = 0.38	Yes: −3.16 No: −2.71 *t* = −0.28 *p* = 0.78	Yes: −3.35 No: −2.93 *t* = 0.20 *p* = 0.85	Yes: −10.3 No: −2.60 *t* = 2.35 *p* = 0.02	Yes: −4.04 No: −2.69 *t* = 0.54 *p* = 0.59

At the 3-month visit, a total of 10 patients experienced a large decline in LVEF greater than 10%. There was no significant difference between these patients and the others in terms of their gender or presence of other CV risk factors (*p* = 0.10–1.0). However, there was a difference in age: participants with a drop in LVEF greater than 10% were 52 ± 14 years of age while the others were 66 ± 11 years of age (*p* = 0.0023). We also found that these patients had a significant decrease in strain absolute values. The patients with a large drop in LVEF had a change in strain of 3.28 ± 2.84 while the others had a change of 1.06 ± 2.94 (*p* = 0.029). There were a total of 5 patients with whose LVEF was less than 53% at the 3-month visit. There was no significant difference between these patients and the others in terms of their gender, CV risk factors, or change in strain values (*p* = 0.27–1.0). We found that patients with a low LVEF (less than 53%) at the 3-month visit were mostly males (*p* = 0.03). Finally, only 2 participants presented at the 3-month visit with a large decline in LVEF greater than 10% to less than 53% and we found no significant difference between these patients and the others (*p* = 0.13–1.0).

## Discussion

The results of this study indicate that an algorithm that provides a method to perform automated determination of mean mid-wall circumferential strain acquired from cine white blood bSSFP images of the LV myocardium is feasible in patients undergoing serial examinations of LV function during receipt of potentially cardio-toxic chemotherapy for the treatment for their cancer. In addition, these assessments of LV circumferential strain using this new technology correlate with subclinical deteriorations in LVEF observed over the first 3 months of patients’ cancer treatment (Fig. 4; Tables 2, 3 and 4). In this patient population, these automated assessments of strain from the cine white blood images were analyzed rapidly (average of 6 min, 47 s), reproducibly, and more frequently than measures of strain acquired with traditional tissue tagging techniques.

Recent consensus statements regarding the application of transthoracic echocardiography (TTE) for patients receiving potentially cardio-toxic chemotherapy have indicated that 15% deteriorations in global measures of LV longitudinal strain are associated with future decrements of LVEF [14]. Toro-Salazar et al. [15] measured global and segmental strain with speckle tracking TTE and CMR strain in childhood cancer survivors exposed to anthracycline therapy. Their study demonstrated the superiority of tagged CMR strain over TTE to identify significant decreases in both global and segmental circumferential and longitudinal strain in subjects with normal indices of global systolic function. Using CMR, deteriorations of LV mean mid-wall circumferential strain obtained with myocardial tissue tagging techniques have been found to associate with future cardiovascular events in the large 6000 participant Multi-Ethnic Study of Atherosclerosis (MESA) cohort and in patients receiving potentially cardio-toxic chemotherapy [3, 16]. However, image quality in cancer patients is not always sufficient often due to “tag fading” and incomplete tracking of the myocardium [4]. The fading of the tags is caused by T1 relaxation and repeated radiofrequency excitation which inherently reduce the signal to noise ratio in the image such that the tracking of the tags at the end of the cardiac cycle becomes very difficult [4]. The methodology incorporated in this study overcomes this limitation by utilizing all of the signal within the image to generate assessments of myocardial strain.

The technique used to measure mean mid-wall circumferential strain in this study differed in terms of the tracking methods from prior studies. Previously, Hor et al. [5] used a method that locally tracked the points on the individual contours of the endocardium and epicardium, while Tsadok et al. [17] proposed to use a non-rigid registration to track these same contours. In these methods, the displacements of pixels inside the LV myocardium (between the endo- and epicardial surfaces) are interpolated rather than calculated. Our method differs in that all pixels in the images are tracked using the non-rigid deformation field allowing for calculation of strain at any pixel that resides within the LV myocardium. In this study, we averaged the pixels at the mid-point between the endocardial and epicardial surfaces to calculate mean mid-wall circumferential strain, the metric that has been previously shown to associate with changes in LV dysfunction after receipt of potentially cardio-toxic chemotherapy.

We did not appreciate a strong correlation (*p* = 0.01) between the strain calculated from the cine images and the strain calculated from the tagged images (Table 3). This could be due to multiple factors. First, in 86.8% of individuals, not all 6 of the myocardial segments were tracked correctly using the tissue tagging process. Therefore, the strain was averaged over the entire myocardium centerline in the cine images while the strain was averaged over only a subset of the 6 segments for tagged images. This results in strain values that are difficult to compare with one another.

A second factor that may have influenced the somewhat poor correlation between the strain obtained from the cine versus tagged images may relate to the fact that the cine automated method of determining strain calculates Lagrangian strain, while the tagged derived measures were reported as Eulerian strain. Lagrangian strain is defined as the lengthening or shortening of the object at the current time instant with respect to the initial length of the object. The Eulerian strain (or natural strain), however, is defined with respect to the infinitesimal amount of deformation during the previous infinitesimal amount of time. Total amount of Eulerian strain is obtained by summing these infinitesimal strains. It has been shown that the Lagrangian strain and the Eulerian strain are non-linearly related. For small deformations, they are almost equal, but for large deformations that occur during cardiac ejection and rapid filling, they can be significantly different [18, 19].

Finally, a third factor that might explain the differences in the cine and tagging derived strain relates to the slice position of the acquired image. While both slices were inherently “middle LV slices,” differences in breath-holding during each respective acquisition could promote a situation in which the slices were acquired in slightly different locations. It is interesting to note that we found a significant correlation between cine derived LV circumferential strain and LVEF (*p* < 0.0001), but no correlation between tagged derived strain and LVEF (*p* = 0.3).

The poor correlation between LVEF and tagged derived strain is reminiscent of previous publications. In [20], Lu et al. study pediatric cancer survivors and show that cine derived strain is more closely correlated to LVEF than tagged derived strain. MacIver et al. [21] cite many clinical studies and have provided a mathematical model indicating that LVEF is determined by both LV myocardial strain and wall thickness, to explain why reduced myocardial strain may be observed in the setting of a normal LVEF.

The multivariable analyses of the association of the cine derived strain and LVEF included age, gender, and cardiovascular risk factors (hypertension, diabetes, CAD, and smoking) as covariates in a general linear model (Tables 5 and 6). After adjusting for these risk factors, the change in cine derived strain associates with the changes in LVEF.

The automated analysis of the cine white blood bSSFP images provided an identical assessment of mean mid-wall LV circumferential strain in 100% of the 142 cases analyzed automatically. In two situations the algorithm failed to properly assess myocardial strain due to an inability of the algorithm to correctly detect the LV blood pool. Because of unusual contrast in the images, the center of the blood pool was not detected on most of the slices, and the blood pool itself was not thresholded appropriately. To handle these two cases, a semi-automated method was utilized. In an average of 6 min and 47 s, the new algorithm calculated strain in all myocardial segments across an average of 14 short axis slices and 2 long axis slices of the left ventricle each with an average of 58 frames in the cardiac cycle. Similar manual contouring of this same volume of data could take over an hour, and as we found in our semi-automated analysis, be less reproducible.

Normal limits for the circumferential strain are very much dependent on the method that is used to calculate the strain. Cheng et al. [22] used tagged CMR and found an average strain at −17.4 ± 2.6 for people less than 65 years of age and −17.0 ± 2.7 for people older than 65. Kleijn et al. [23] found a value of −30.6 ± 2.6 with 3D echocardiography. Andre et al. [24] found a value of −21.3 ± 3.3 with cine CMR feature tracking. Marwick et al. [25] found a value of −18.6 ± 0.1 with 2D echocardiography. The values differ between different studies because different calculation methods are used. This is the reason why the echocardiography community is trying to define a standard [26]. The normal limits for this new method have not been determined yet. This issue is similar to that found in TTE where methods to measure strain associated with different vendors provide different values [14]. While monitoring patients being treated for cancer with potentially cardio-toxic chemotherapy, it is more important to appreciate decrements in strain values that occur longitudinally over time during the course of the treatment. The results of this study demonstrate that changes in measures of cine derived strain associate with decrements in LVEF after receipt of potentially cardio-toxic chemotherapy (*p* < 0.0001).

The addition of easily measured LV mean mid-wall circumferential strain to the CMR evaluation of patients treated for cancer may help identify those at risk and potential mechanisms of developing heart failure after cancer treatment. Currently, CMR assessments of LV volumes, T1 mapping, LV myocardial extracellular volume fraction, and aortic stiffness have been found abnormal in patients treated for cancer [3]. Developing methods such as the one described in this study that reduces analysis time can provide both researchers and clinicians with LV strain measures without adding to the image acquisition time. In TTE, assessments of myocardial strain are described to deteriorate before decrements of LVEF are appreciated [14]. In addition, assessments of strain can be used to confirm small changes in LVEF that are appreciated during the clinical study.

There are limitations to the current study. First, the methodology described and implemented in this study is not yet widely available clinically. Further development will be required to make the strain assessments available for clinical application. Second, the sample point in this study was 3 months into the administration of potentially cardio-toxic chemotherapy. This time interval is one in which several clinical management strategies suggest monitoring of LV function to ensure absence of cardiovascular injury upon receipt of potentially cardio-toxic chemotherapy [14]. Importantly however, the clinical relevance of these early changes in myocardial strain or LV performance is not well understood. Further studies are necessary to understand the utility of this methodology for predicting changes in LVEF that may occur as a result of receipt of potentially cardio-toxic chemotherapy. Our cohort will also be receiving a CMR exam 2 years after the first dose of chemotherapy. We are still in the process or acquiring and analyzing these data.

## Conclusions

In conclusion, we have proposed an automated method to measure LV mean mid-wall circumferential strain from cine white blood bSSFP in a clinical setting for patients receiving treatment for cancer with potentially cardio-toxic chemotherapy. The method to calculate strain is fully automatic and requires no user interaction. The results of this study indicate that changes in LV mean mid-wall circumferential strain obtained from the analysis of these cine images associate with changes in LVEF during the first 3 months of chemotherapy. The cine derived strain correlates with subclinical deteriorations in LVEF after accounting for other risk factors associated with cardiovascular events. These measures of LV mean mid-wall circumferential strain are reproducible, and do not require an extra image acquisition since they are derived from the same images used to calculate LV volumes, LVEF and mass. Future development of this technology could enhance transition of LV strain measures into clinical examinations of the heart performed in patients receiving treatment for cancer.

## Abbreviations

ANOVA: Analysis of Variance
CAD: Coronary Artery Disease
CMR: Cardiovascular Magnetic Resonance
CV: Cardiovascular
ED: End Diastole
EDV: End Diastolic Volume
EF: Ejection Fraction
ES: End Systole
ESV: End Systolic Volume
FOV: Field Of View
HARP: Harmonic Phase Analysis
LA: Long Axis
LV: Left Ventricle
MLHFQ: Minnesota Living with Heart Failure Questionnaire
SA: Short Axis
bSFFP: Balanced Steady State Free Precession
TE: Echo Time
TR: Repetition Time
TTE: Transthoracic echocardiography
